# CEACAM1-4L Promotes Anchorage-Independent Growth in Melanoma

**DOI:** 10.3389/fonc.2015.00234

**Published:** 2015-10-19

**Authors:** Stefanie Löffek, Nico Ullrich, André Görgens, Florian Murke, Mara Eilebrecht, Christopher Menne, Bernd Giebel, Dirk Schadendorf, Bernhard B. Singer, Iris Helfrich

**Affiliations:** ^1^Skin Cancer Unit of the Dermatology Department, Medical Faculty, West German Cancer Center, University of Duisburg-Essen, Essen, Germany; ^2^German Cancer Consortium (DKTK), Medical Faculty, University of Duisburg-Essen, Essen, Germany; ^3^Institute for Transfusion Medicine, Medical Faculty, University of Duisburg-Essen, Essen, Germany; ^4^Institute of Anatomy, Medical Faculty, University of Duisburg-Essen, Essen, Germany

**Keywords:** CEACAM1, melanoma, anchorage-independent cell growth, metastatic potential, heterogeneity

## Abstract

Widespread metastasis is the leading course of death in many types of cancer, including malignant melanoma. The process of metastasis can be divided into a number of complex cell biological events, collectively termed the “invasion-metastasis cascade.” Previous reports have characterized the capability of anchorage-independent growth of cancer cells *in vitro* as a key characteristic of highly aggressive tumor cells, particularly with respect to metastatic potential. Biological heterogeneity as well as drastic alterations in cell adhesion of disseminated cancer cells support escape mechanisms for metastases to overcome conventional therapies. Here, we show that exclusively the carcinoembryonic antigen-related cell adhesion molecule 1 (CEACAM1) splice variant CEACAM1-4L supports an anchorage-independent signature in malignant melanoma. These results highlight important variant-specific modulatory functions of CEACAM1 for metastatic spread in patients suffering malignant melanoma.

Metastatic melanoma is a devastating disease of increasing incidence. Tumor progression starts very early in this disease, resulting in a median survival of 6–12 months in patients with advanced melanoma (1). Melanoma cell metastasize predominantly via tumor-associated lymphatic vessels to regional lymph nodes, and subsequently, lymph node metastasis is a major determinant for the staging and clinical management of melanoma (2). Thus, the main cause of death in melanoma patients is widespread metastases that show often resistance to current therapies. To this end, extensive efforts have been made to understand the cellular and molecular processes underlying the metastatic cascade for further development of therapeutic approaches directed against disseminated disease. The first step of the metastatic cascade occurs in the primary tumor, where subpopulations of cancer cells lose their cell–cell contact, exit the tumor mass and invade locally through the extracellular matrix and tumor-associated stromal cell layers. Subsequently, cancer cells intravasate into the microvasculature of the blood or lymphatic system, survive in the circulation, extravasate into the parenchyma of distant organs and adapt to the foreign microenvironment in order to form metastases (3). Dysregulation of cell adhesion molecules has already been associated with disease progression in malignant melanoma (4). In this context, evidence has amassed that expression of the multi-functional cell–cell adhesion protein CEACAM1 may be involved in the multistep process of metastatic spread in melanoma (5). CEACAM1, a transmembrane protein of the CEA family within the immunoglobulin superfamily (6), has been shown to be expressed in various human epithelial (7, 8), activated endothelial (9), and on a variety of hematopoietic cells (10, 11). Mainly four CEACAM1 isoforms are known to be co-expressed in human tissue with either three (CEACAM1-3) or four (CEACAM1-4) highly glycosylated extracellular Ig-like domains, a single-pass transmembrane domain and either a short (S) or long (L) cytoplasmic tail (12). It has already been described that the long cytoplasmic domain, in contrast to the short version, contains two ITIM motifs, which play an important role for the initiation of cellular signaling (13). CEACAM1 has been controversially discussed as tumor suppressor but also as driver for invasion in different tumor entities (14, 15). Loss or lower level of CEACAM1 expression has been detected in colon (16), prostate (17), and breast cancers (18), whereas high expression levels have been found in adenocarcinomas (19), non-small lung cancers (20), and melanoma (21). In addition, the CEACAM1 expression in confluent, contact-inhibited, and proliferating epithelial cells differs significantly with respect to the amount and isoform ratio (22, 23). Beside the membrane anchored CEACAM1 variants, also soluble and microvesicle-bound versions were described (24, 25). Interestingly, the CEACAM1 expression levels in biopsies (26) and concentrations of soluble CEACAM1 in sera (27, 28) of melanoma patients have been reported as a strong clinical predictor of poor prognosis and high risk of metastasis. In consequence, recent studies discuss CEACAM1 as a novel diagnostic and therapeutic target in patients of malignant melanoma.

Recent studies identified the long cytoplasmic domain of CEACAM1 (CEACAM1-L) as driver for invasion in hepatocellular carcinoma (29) and colon cancer (30). Nevertheless, most studies in melanoma were focused on overall CEACAM1 without distinguishing the different isoforms. Generating transfectants explicitly expressing just a single splice variant of CEACAM1 in the human melanoma cell line, Ma-Mel-86a (Figures S1A,B in Supplementary Material) we recently identified, that CEACAM1 expression impacts melanoma progression and antitumor immunity in a variant-specific mode of action (31). Previous reports identified cancer cells with metastatic potential by their ability to exhibit anchorage-independent growth (colony forming capacity in semisolid media), a phenotype, which has also been described as a crucial marker for *in vitro* transformation (32). Since studies exploring the impact of the four different CEACAM1 isoforms within the context of metastatic cancer cell dissemination are completely missing, we now validated the signature of each splice variant with respect to their colony forming capacity. Strikingly, we could detect variant-specific changes in the capacity to grow *in vitro* under anchorage-independent conditions by analyzing the CEACAM1 isoform transfectants, whereas expression of exogenous CEACAM1-3S leads to initial tumor cell assembling in semisolid media but diminished the formation of proliferative colonies, indicated by the reduced colony size (Figures 1A,B). Furthermore, expression of CEACAM1-4S significantly dampens the number of colonies (Figure 1B), while CEACAM1-3L does not affect this phenotypic signature. Interestingly, among all isoforms, only CEACAM1-4L expression results in a significant increase in colony size when compared to the empty vector control (Figure 1B) while the total number of colonies was not altered. In order to exclude off-target-effects caused by the expression of CEACAM1-4L, we performed a RNAi approach in pCL6-CEACAM1-4L-IRES-eGFP (pCL6-CC1-4L-IEG) over-expressing cells using control and CEACAM1 specific shRNA (for details, please see Materials and Methods). CEACAM1 over-expression and the knock-down were confirmed by Western Blot analysis (Figure 1C). As expected, the mean colony size was significantly reduced when cells where grown in a soft agar assay, indicating a crucial rule of CEACAM1-4L in anchorage-independent growth in malignant melanoma, which was further verified by the knock-down of endogenous CEACAM1 in UKRV-Mel-15a cells (Figure 1D). As metastatic spread is associated with loss of adhesion, we performed adhesion assays using lentiviral-induced expression of pCL6-CEACAM1-4L-IRES-eGFP (pCL6-CC1-4L-IEG) in UKE-Mel-1a cells, a further cell line without endogenous CEACAM1-expression. In line with the increase in anchorage-independent growth pCL6-CC1-4L-IEG expression revealed reduced adhesion to collagen I when compared to mock control cells (pCL6-IEG) and this effect was reversed by the knock-down of CEACAM1 (Figure 1E). Together these data indicate a crucial role of CEACAM1-4L in the initiation of metastatic processes in malignant melanoma.

**Figure 1:**
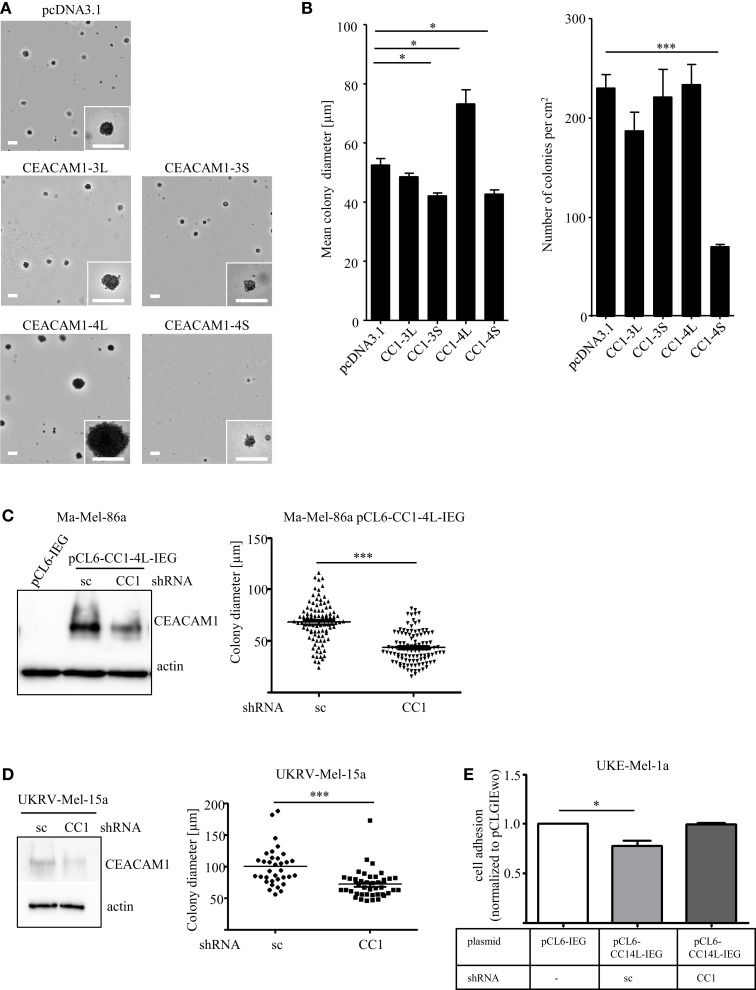
**(A,B)** Influence of CEACAM1 splice variants on anchorage-independent growth of melanoma cells. The cell line Ma-Mel-86a was transfected with empty vector (pcDNA3.1), CEACAM1-3L (CC1-3L), CEACAM1-3S (CC1-3S), CEACAM1-4L (CC1-4L), and CEACAM1-4S (CC1-4S) and cultured in soft agar for 14 days. **(A)** Representative images are shown. Scale bar, 100 μm. **(B)** Quantitative assessment for the number and diameter of colonies formed by CEACAM1 isoform transfectants. Colonies in the area of 1 cm^2^ were measured. Shown are mean values of three independent experiments. **(C)** Ma-Mel-86a cells were transduced with viral particles containing either the empty vector pCL6-IRES-eGFP (pCL6-IEG) or pCL6-CEACAM1-4L-IRES-eGFP (pCL6-CC1-4L-IEG). pCL6-CC1-4L-IEG expressing cells were further transfected with scrambled (sc) or CEACAM1 (CC1) specific shRNA. Stable expression of pCL6-CC1-4L-IEG was analyzed by Western Blot (left panel). Beta-actin was used as loading control. Ma-Mel-86a pCL6-CC1-4L-IEG cells transfected with sc or CC1 shRNA were cultured in soft agar for 7 days. Graph shows quantitative assessment of colony diameter. **(D)** UKRV-Mel-15a cells were transfected with sc or CC1 shRNA. Expression of endogenous CEACAM1 was analyzed by Western Blot (left panel). Beta-actin was used as loading control. These cells were cultured in suspension for 5 days. Graph shows quantitative assessment of colony diameter. *p < 0.05, ***p < 0.001, **(E)** UKE-Mel-1a cells were transduced with viral particles containing either pCL6-IRES-eGFP (pCL6-IEG) or pCL6-CEACAM1-4L-IRES-eGFP (pCL6-CC1-4L-IEG). pCL6-CC1-4L-IEG expressing cells were further transfected with scrambled (sc) or CEACAM1 (CC1) specific shRNA. All cell lines were allowed to adhere to collagen I for 45 min.

Although multiple genetic factors for anchorage independency have been identified so far (32, 33), the detailed molecular signature for this phenotypic behavior is largely unknown. To our understanding, tumor cells identified for their highly aggressive signature, e.g., anchorage-independent growth, should also present a highly invasive phenotype which has been shown to correlate with changes in the tumor secretome. Consequently, we used a Proteome Profiler Antibody Array to analyze changes in the expression of soluble factors in all CEACAM1 isoform transfectants. This analysis revealed distinct and significant changes in the expression of factors associated with matrix metalloproteinases (MMP) expression and activation, including uPAR, RANTES, IL-6, and EMMPRIN, in particular in CEACAM1-4L expressing cells (34–37) (Figure 2A). In contrast, some other factors including e.g., the Macrophage migration inhibitory factor (MIF; mainly involved in immune response) and the Wnt-signaling involved factor Dickkopf-1 (DKK1), are almost equally up-regulated by the expression of all CEACAM1 isoforms when compared to cells lacking CEACAM1 expression. It has been well established that the invasive phenotype of melanoma cells, is associated with the expression of MMPs. In particular, the gelatinase MMP-2 and their endogenous inhibitors, the tissue inhibitor of MMPs (TIMPs), drive degradation of stromal compartments for cancer cell invasion (38, 39). Activation of proMMP-2 (the inactive form of MMP-2) occurs primarily via the formation of a tri-molecular complex consisting of the membrane bound MMP-14, a protease already characterized for their impact on colony formation (40), TIMP-2 and MMP-2 (41, 42). In this regard, we were not surprised to detect significant induction of MMP-2 and MMP-14 mRNA expression in the CEACAM1-4L transfectants (Figure 2B). In accordance with these findings, high levels of MMP-14 resulted in enhanced cleavage and proteolytic activity of MMP-2 in the human Ma-Mel-86a melanoma cell line expressing CEACAM1-4L (Figure 2C), which in consequence, drives these cells to grow under anchorage-independent conditions. In line, CEACAM1-3S and CEACAM1-4S transfectants, which failed to generate growing colonies in semisolid media, possessed decreased MMP-2 activity (Figure 2C). In order to analyze the functional involvement of MMPs in the anchorage-independent proliferation, we grew Ma-Mel-86a CEACAM1-4L transfectants in the presence or absence of MMP-inhibitors. Indeed, treatment with the MMP-2 specific inhibitor ARP 100 resulted in significant reduction of the mean colony size (Figure 2D), indicating that the increase in anchorage-independent proliferation partially depends on the activation of pro-MMP-2 in the presence of the CEACAM1-4L splice variant. However, since the broad MMP inhibitor, Marimastat showed potential to enhance this effect, we conclude that also further MMPs influence this cellular behavior and are connected to the interplay with CEACAM1-4L.

**Figure 2:**
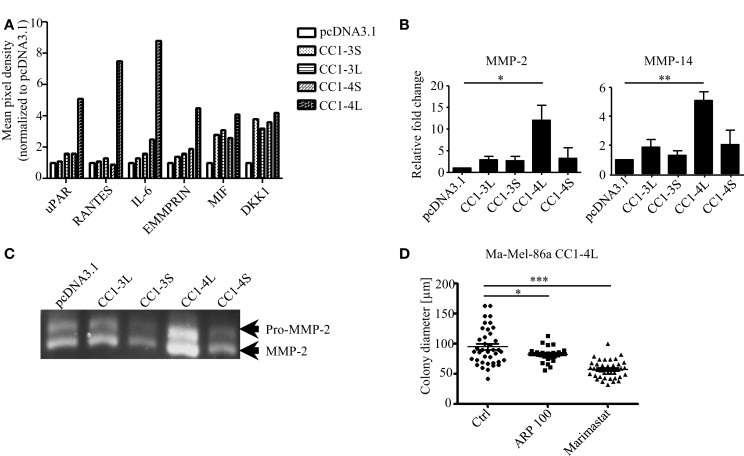
**Involvement of MMPs in anchorage-independent growth of CC1-4L transfectants**. **(A)** Analysis of factors secreted into the medium by CEACAM1 isoform expressing melanoma cells. Conditioned media were prepared as described in Section Materials and Methods and applied to a Proteome Profiler Antibody Array. Graph shows the quantification of indicated soluble factors as mean pixel density quantified using ImageQuant software. Data were normalized to the signals of the pcDNA3.1 transfectants. **(B)** CEACAM1 splice variant transfectants were analyzed for MMP-2 and MMP-14, expression by qRT-PCR. Shown are mean values of three to four independent experiments. *p < 0.05, **p < 0.01. **(C)** Detection of MMP-2 activity in supernatant obtained from indicated CEACAM1 splice variant transfectants by gelatin zymography. Equal amounts of conditioned media (normalized to cell number) were separated on SDS-gelatin zymography. The inactive (pro-) and active forms of MMP-2 are depicted on the right. One experiment out of three independent analyses is shown. **(D)** Ma-Mel-86a CC1-4L expressing cells were cultured 2 days in the presence of DMSO (Ctrl), MMP-2-specific inhibitor (ARP 100) (13 nM), or Marimastat (2 μM) before embedding into soft agar. DMSO and inhibitors were inoculated into the top agar and added to the covering medium. Medium was changed every second day. Single cells were allowed to form colonies for 7 days. Graph shows quantitative assessment of colony diameter. *p < 0.05, ***p < 0.001.

Taken together, we showed that CEACAM1 splice variants act in an antagonistic fashion by modulating phenotypic signatures involved in metastatic dissemination of melanoma cells. These observations highlight the significance of the variant-specific function of CEACAM1 for melanoma progression. Nevertheless, our findings warrant further mechanistic analysis to focus on the molecular crosstalk between CEACAM1 and molecules, known to be involved in the metastatic cascade of melanoma cells. This may offer new insights for novel therapeutic approaches in melanoma patients.

## Material and Methods

### Cells and Cell Culture

The cell line Ma-Mel-86a and UKE-Mel-1a were established from nodal metastasis and UKRV-Mel-15a generated by using a skin metastasis of stage III melanoma patients [according to the AJCC criteria (43)].

All cell lines were provided by the Skin Cancer Biobank (SCABIO) of the Dermatology Department, University Hospital Essen, Germany. Patient informed consent was obtained and studies were approved by the Institutional Review Board. Clinical information such as age, gender, stage of disease, and survival time were documented and retrieved from the database (Achiver Anyware Medical, Achiver Software). RPMI 1640 (Ma-Mel-86a and UKRV-Mel-15a) or DMEM (UKE-Mel-1a) was supplemented with 10% fetal calf serum (FCS), 1% Penicillin/Streptomycin, and 1% l-Glutamine (all from PAA Laboratories) and used for cultivation at 37°C and 5% CO_2_. Cells were regularly tested for mycoplasma contamination.

### Reagents

The MMP-2 specific inhibitor ARP 100 was obtained from Santa Cruz. Marimastat was obtained from Calbiochem. All compounds were applied in the indicated concentrations.

### Plasmid Constructs and Transfection

The coding region of human CEACAM1-3L (NM_001184813.1), CEACAM1-3S (NM_001184816.1), CEACAM1-4L (NM_ 001712.4), and CEACAM1-4S (NM_001024912.2) were cloned into the pcDNA3.1(−) Neo expression vector (Invitrogen) and verified by sequencing. Constructs were transfected into the Ma-Mel-86a cell line using Metafectene (Biontex) according to manufacturer’s protocol. Afterward, single clones were established and selectively grown in medium containing 1mg/ml G418 (Carl Roth). For construction of lentiviral constructs with eGFP reporters, the coding region of the CEACAM1-4L isoform was subcloned into pCL6-IRES-EGwo plasmids (44) via *Xho*I and *Eco*RI.

### Lentiviral Transduction

Lentiviral supernatants were generated as described previously (45). Briefly, HEK293T cells were co-transfected with either pCL6-IRES-eGFPwo (pCL6-IEG, control) or pCL6-CEACAM1-4L-IRES-eGFPwo plasmids (pCL6-CC1-4L-IEG), together with the pCD/NL-BH helper plasmid (46) and the codon-optimized, human foamy virus envelope expression plasmid pcoPE (47) using Jetpei (Polyplus, Illkirch Cedex, France) transfection reagent according to the manufacturer’s recommendations. Gene expression from the human cytomegalovirus (CMV) immediate-early gene enhancer/promoter was induced 24 h after transfection with 10 mM sodium butyrate. Forty-eight hours after transfection, supernatants were collected, filtered through 0.45-μm filters (Sartorius), concentrated by centrifugation at 25,000 × *g* for 90 min at 4°C and stored at −80°C. Virus stocks were titrated on HEK293T cells before use.

Ma-Mel-86a and UKE-Mel-1a cells were transduced by overnight exposure to virus stocks, passaged at least twice, and subsequently sort-purified (eGFP expression) on a BD FACSAria IIIu cell sorter. The established cell line was passaged at least 5 times before experiments were performed.

### CEACAM1 shRNA

Knock-down of either endogenous CEACAM1 in UKRV-Mel-15a cells or of over-expressed pCL6-CEACAM1-4L-IRES-eGFP (pCL6-CC1-4L-IEG) in Ma-Mel-86a and UKE-Mel-1a cells was performed by transfecting SureSilencing shRNA Plasmid (Hs.512682) (Quiagen) using jetPRIME transfection reagent (Polyplus) according to the manufacturer’s recommendations. Negative control shRNA vector (scrambled artificial sequence) was used as control (Quiagen). To generate stable clones lacking CEACAM1 expression, transfected melanoma cells were selected in 1 mg/ml of G418.

### RT-PCR and qRT-PCR

Total RNA was isolated and c-DNA was synthesized as described before in Ref. (48). All semi-quantitative RT-PCRs were performed in linear range of amplification and 1 μg cDNA was used as template. Following primers were utilized: Actin forward 5′-ACCCTGAAGTACCCCAT-3′, reverse 5′-TAGAAGCATTTG CGGTG-3′; CEACAM1 splice variants forward 5′-AACCAAA GCGACCCCATCA-3′, reverse 5′-RTGGGTCATTGGAGTGG TCC-3′. Isoform specific amplification products (bp): CEACAM1-4L = 779; CEACAM1-4S = 726; CEACAM1-3L = 491 bp; CEACAM1-3S = 438. Quantitative RT-PCR was performed using specific TaqMan Gene Expression assays in combination with the StepOnePlus Real-Time PCR system (Applied Biosystems). Relative fold change was calculated by the 2^−ΔΔCt^ method after normalizing the Ct values to GAPDH. ΔCt-values were used for statistically evaluation.

### Western Blot

Proteins were extracted from melanoma cells using Cell Lysis Buffer^®^ (Cell Signaling) and separated by SDS-PAGE. Proteins were transferred onto nitrocellulose membranes and probed with mouse anti-human Actin (MP Biomedicals) and mouse anti-human CEACAM1 (clone 4/3/17, Aldevron). Membranes were probed with appropriated secondary antibodies conjugated to horseradish peroxidase (HRP) (Dianova) and visualized by Pierce ECL Western Blotting Substrate (Thermo Scientific).

### Colony Forming Assay in Soft Agar and Suspension

Contact independent cell growth and colony formation were tested by seeding cells in 0.2% sterile agarose/RPMI/FCS on top of 0.5% sterile Noble agar/RPMI/FCS (bottom layer) as described before (48). In brief, 1 ml of 1% Noble Agar (BD Biosciences) mixed with equal volume of 2× RPMI was added to a 6-well plates and incubated at room temperature for 30 min. 2.5 × 10^3^ cells were re-suspended in 2× RPMI, mixed 1:1 with 0.4% agarose (41°C) (Bio-Budget), and seeded on top of the solidified Noble-Agar. Five hundred microlitre culture medium was added after solidification and replaced with fresh medium every week. Each experiment was performed in triplet. One centimeter square was photographed utilizing Zeiss microscope (Zeiss AxioObserver.Z1), number and diameter of colonies were determined by using ImageJ software.

UKRV-Mel-15a cells (these cells do not form proper colonies in soft agar) were cultured in medium for 5 days. Cell adhesion to the culture plate was prevented by growing the cells on top of 0.5% Noble Agar bottom layer (as described above).

### Proteome Profiler Antibody Array

Serum-free conditioned media from CEACAM1 isoform expressing Ma-Mel-86a melanoma cells were analyzed using a Proteome Profiler Antibody Array (Human XL Cytokine Array Kit R&D Systems) according to the manufacturer’s instructions. Briefly, membranes were incubated overnight at 4°C in melanoma cell-conditioned medium, washed twice and incubated 1 h at room temperature with a mixture of biotin-conjugated antibodies. After incubation, membranes were washed twice and incubated with HRP-conjugated antibodies. Bound secondary antibodies were detected by enhanced chemiluminescence (ECL) reaction. Quantification of signal intensities was performed using ImageQuant software.

### Adhesion Assay

UKE-Mel-1a cells were seeded onto 96-well plates coated with collagen I (10 μg/ml) (Sigma). Uncoated areas were blocked with 1%BSA/PBS for 30 min. Cell adhesion was allowed to proceed for 45 min. Non-adherent cells were removed by washing with PBS. Adherent cells were fixed with methanol and stained with 0.5% crystal violet in 20% (*v*/*v*) methanol. The dye was released from cells by addition of 1% SDS, and the absorbance of the dye solution determined at 540 nm. The adhesion of the cells was expressed as a percentage relative to mock transfectants.

### Gelatin Zymography

Serum-free conditioned media were separated by gelatin zymography as previously described (49). In brief, cells were grown in FCS-free media for 24 h. Volumes of conditioned media were normalized to cell number and fractioned on 10% SDS-PAGE containing 1 mg/ml gelatin (AppliChem). After electrophoreses, gels were washed in 2.5% Triton-X 100 for 45 min and incubated overnight in substrate buffer (50 mM Tris–HCl, pH 8.0, 5 mM CaCl_2_). Gels were stained with Coomassie (BioRad).

### Statistical Analysis

All results were shown as mean ± SEM. Student’s *t*-tests were used to derive (two-sided) *p*-values for comparisons between experimental groups. We applied a two-sided significance level α of 5%. All calculations were performed by using the Graph Pad Prism software (GraphPad software Inc., San Diego, CA, USA).

## Author Contributions

SL, NU, BS, and IH substantially contributed to the conception of the work; SL, NU, AG, FM, ME, CM, BG, BS, and IH designed the work and analyzed data; SL, NU, BS, and IH drafted the work for important intellectual content and wrote the manuscript; SL, BS, DS, and IH finally approved the manuscript for publication.

## Conflict of Interest Statement

The authors declare that the research was conducted in the absence of any commercial or financial relationships that could be construed as a potential conflict of interest. The Guest Associate Jenny Landsberg declares that, despite having published with authors Dirk Schadendorf and Iris Helfrich, the review process was handled objectively and no conflict of interest exists.
