# Supplementary figures and images for: CEACAM1-4L Promotes Anchorage-Independent Growth in Melanoma
Source: Front Oncol. 2015 Oct 19;5:234. doi: 10.3389/fonc.2015.00234 (PMC4609850; doi:10.3389/fonc.2015.00234)

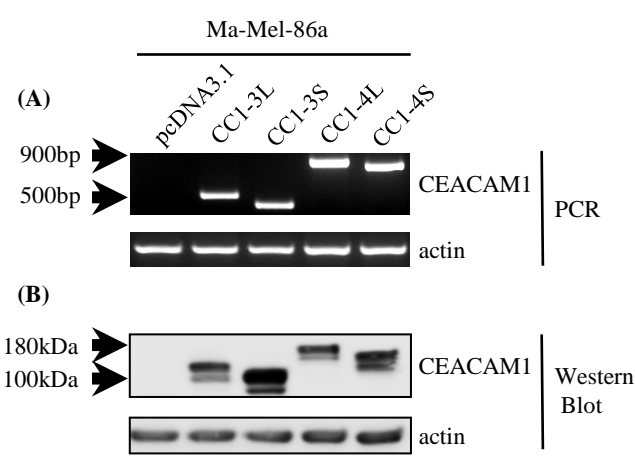

Supplement: Supplementary file 1 [file Image_1.PDF]
